# Differential Role of Anti-Viral Sensing Pathway for the Production of Type I Interferon β in Dendritic Cells and Macrophages Against Respiratory Syncytial Virus A2 Strain Infection

**DOI:** 10.3390/v11010062

**Published:** 2019-01-15

**Authors:** Dong Sun Oh, Tae Hoon Kim, Heung Kyu Lee

**Affiliations:** 1 Graduate School of Medical Science and Engineering, Korea Advanced Institute of Science and Technology (KAIST), Daejeon 34141, Republic of Korea; dongsun.oh@kaist.ac.kr (D.S.O.); thkim21@cha.ac.kr (T.H.K.); 2 Department of Internal Medicine, CHA Bundang Medical Center, CHA University, Seongnam 13496, Republic of Korea; 3 KAIST Institute for Health Science and Technology, KAIST, Daejeon 34141, Republic of Korea

**Keywords:** Respiratory syncytial virus, TLR7, MyD88, MAVS, Type I interferon, Dendritic cell, Macrophage

## Abstract

Respiratory syncytial virus (RSV) is a major cause of respiratory infectious disease in infants and young children. Dendritic cells (DCs) and macrophages (MACs) are known to play important roles in RSV recognition, and in the production of type I interferons (IFNs) and pro-inflammatory cytokine in RSV infection. Toll-like receptor 7 (TLR7), myeloid differentiation primary response 88 (MyD88), and mitochondrial antiviral-signaling protein (MAVS) are known to be important for the RSV sensing pathway in DCs and MACs. However, despite the critical roles of type I IFNs in the anti-RSV immune response, the pattern recognition receptors (PRRs) that are required for RSV sensing in DCs and MACs remain unclear. Here, we investigate the pathway activated by RSV A2 strain infection using an IFN-β/YFP reporter mouse model to visualize IFN-β-producing cells and in vitro RSV infection in bone marrow-derived DCs (BM-DCs) and macrophages (BM-DMs). We present our finding that MyD88, but not TLR7, are important for RSV recognition and type I IFN and pro-inflammatory production in DCs and MACs. MAVS-deficient BM-DCs and BM-DMs show impaired induction of IFN-β production upon RSV stimulation, and this effect is RSV replication-dependent. Our study provides information on cell type-specific PRR requirements in innate immune responses against RSV infection.

## 1. Introduction

Respiratory syncytial virus (RSV) is a leading cause of respiratory disease in infants and young children, causing recurrent childhood wheezing or asthma [1,2]. Most infants are infected with RSV at least once within the first 2 years of life; many suffer RSV-induced pathogenicity and may suffer repeated infections throughout life [3]. Although many attempts have been made to develop an RSV vaccine, no vaccine has been approved to date [4]. 

Type I interferon (IFN) production is an immediate innate immune response to viral infection, and type I IFNs play an important role in the antiviral response to RSV infection [5]. Although RSV has evolved escape strategies to maintain infectivity during type I IFN-induced antiviral responses, several reports have shown that RSV can induce type I IFN production [6]. A number of studies have suggested that dendritic cells (DCs) and macrophages (MACs) can secrete type I IFNs when infected with RSV [7]. However, the identification of cell types that can secrete type I IFNs during RSV infection and reliable verification of this secretion are required.

RSV infection or uptake by resident MACs and DCs incites a direct antiviral response through cytokines and chemokines [8]. These cells have pattern recognition receptors (PRRs), Toll-like receptors (TLRs), and retinoic acid-inducible gene-I (RIG-I)-like receptors (RLRs), which recognize pathogen-associated molecular patterns (PAMPs) on the pathogen [9]. These immune cells have different levels of PRRs, and the cell type-specific expression of PRRs plays a unique role in the innate immune response. In DCs and MACs, TLRs are required for RSV sensing, initiation of type I IFN production, and pro-inflammatory cytokine secretion; other reports also show that the RIG-I/mitochondrial antiviral-signaling protein (MAVS) pathway has an important role in the innate immune response to RSV infection [10,11,12]. Therefore, it is important to investigate these claims and determine which pathway is the most important in RSV infection.

Here, we investigated the cellular production of IFN-β in RSV A2 strain (hereafter referred to as RSV), one of the major strain of RSV, infection by using an IFN-β/YFP reporter mouse, a credible and unbiased tool for the visualization and spatiotemporal tracking of IFN-β-producing cells. Further, we determined the signaling pathway activated by RSV to produce type I IFN and proinflammatory cytokines. We found that myeloid differentiation primary response 88 (MyD88) and MAVS, but not TLR7, are important for RSV recognition and type I IFN production in DCs and MACs.

## 2. Materials and Methods

### 2.1. Animals

IFN-β/YFP reporter (B6.129-Ifnb1tm1Lky/J) [13], MyD88-deficient (B6.129P2(SJL)-Myd88tm1.1Defr/J) [14], TLR7-deficient (B6.129S1-Tlr7tm1Flv/J) [15], and MAVS-deficient (B6.129S1-Tlr7tm1Flv/J) [16] mice have been described previously. TLR7 and MAVS-deficient mice were purchased from The Jackson Laboratory. IFN-β/YFP reporter mice, provided by Suk-Jo Kang (KAIST), were crossed with TLR7-deficient, MyD88-deficient, or MAVS-deficient mice. All mice were bred in a specific pathogen-free facility at Korea Advanced Institute of Science and Technology (KAIST), and all procedures were in accordance with the guidelines and policies for rodent experimentation provided by the Institutional Animal Care and Use Committee (IACUC) of KAIST. This study was approved by the IACUC of KAIST (KA2013-55, October, 24, 2013). 

### 2.2. Mice Genotyping

To isolate genomic DNA and check genotype of mice, small pieces of mice tail were digested with tail digestion buffer (10 mM Tris (Welgene), 50 mM KCl (Welgene), 2.5 mM MgCl_2_ (Welgene), 0.45% NP-40 (Bio-Basic), 0.45% Tween-20 (Amresco), and 0.2 mg/mL Proteinase K (AG Scientific) in 3’dilstiled water). Oligonucleotides for genotyping were purchased from Bioneer and genotype was determined by PCR using specific primer as listed follow. IFN-β/YFP reporter (wild type forward – 5’ CCC TAT GGA GAT GAC GGA GAA GAT GC 3’, common – 5’ CAA TCC CAT AGC AGG TGA TGC C 3’, mutant forward – 5’ TGG TCC TGC TGG AGT TCG TGA CCG C 3’); MyD88-deficient (wild type forward–5’ TGG CAT GCC TCC ATC ATA GTT AAC C 3’, common – 5’ GTC AGA AAC AAC CAC CAC CAT GC 3’, mutant forward – 5’ TGG CAT GCC TCC ATC ATA GTT AAC C 3’); TLR7-deficient (wild type forward - 5’ AGG GTA TGC CGC CAA ATC TAA AG 3’, common – 5’ ACC TTT GTG TGC TCC TGG AC 3’, mutant forward – 5’ TCA TTC TCA GTA TTG TTT TGC C 3’); MAVS-deficient (wild type forward - 5’ AGC CAA GAT TCT AGA AGC TGA GAA 3’, common – 5’ TAG CTG TGA GGC AGG ACA GGT AAG G 3’, mutant forward – 5’ GTG GAA TGT GTG CGA GGC CAG AGG C 3’).

### 2.3. RSV Propagation

The A2 strain of RSV was kindly provided by Dr. Jun Chang (Ewha Womans University). RSV was grown in HEp-2 cells and titrated for infectivity [17,18]. In detail, MOI = 0.01 of RSV was infected with mono-layers of HEp-2 cell culture in 40 mL of 10% fetal bovine serum (FBS; Hyclone) containing DMEM (Welgene) in T-175 flask (SPL). When extensive syncytia were observed around 4 to 5 days after RSV infection, viruses contained cell-culture supernatants were harvested and concentrated at 23,000 rpm of ultra-centrifugation for 45 min in 4 °C. The RSV pellet were re-suspended in Dulbecco’s Phosphate-Buffered Salines (DPBS) (Welgene). RSV titer was determined using an RSV plaque assay [19]. In detail, 400 μL of serial 10-fold diluted RSV in serum-free DMEM were infected with mono-layers of HEp-2 cell cultures in 6-well plate (SPL) for 2 h and incubated at 37 °C with shaking every 15 min. Medium were removed after 2 h of infection, 3 mL of 2% agarose in 10% FBS containing DMEM were added and incubated for 5 days. Cells were then fixed with 1% formaldehyde in DPBS and solidified agarose gels were removed. Next, Cells were stained with 0.05% of natural red (Sigma Aldrich) and RSV plaques were counted. For the inactivation of RSV, inactivated RSV was achieved by heating virus at 95 °C for 15 min. RSV stock in DPBS was stored at -80 °C without sucrose. To maintain RSV infectivity, we titrated RSV virus stocks again before the related experiments.

### 2.4. RSV Infection In Vitro and Cytokine Measurements

Granulocyte-macrophage colony-stimulating factor (GM-CSF)-derived bone marrow-derived dendritic cells (BM-DCs) or macrophage colony-stimulating factor (M-CSF)-derived bone marrow-derived macrophages (BM-DMs) were prepared as previously described [20]. To isolate bone marrow (BM) cells in mice, femurs and tibiae were removed form mice and bone marrow were isolated by using 10 mL syringe with 10 mL of serum-free DMEM. Next, BM cells were counted with hemo-cytometer after red blood cell lysis with ACK lysis buffer (150 mM NH_4_Cl, 10 mM KHCO_3_, 0.1 mM Na_2_EDTA in 3’DW). BM-DCs were generated by incubating 1 × 10^6^ BM cells with 20 ng/mL GM-CSF-supplemented 1 mL per well of RPMI 1640 media (Welgene) containing 10% FBS and 1% of penicillin/streptomycin (Welgene) in 24 well cell culture plate (Corning) for five days. In every other day, 500 μL of GM-CSF contained media was supplemented. To harvest BM-DCs, loosely adherent cells were harvested by pipetting. BM-DMs were generated by incubating 10 × 10^6^ BM cells with 10 mL of 30% of in house prepared (seven days of cell culture supernatants of M-CSF secreting L929 cell line) M-CSF-supplemented RPMI 1640 media containing 10% FBS and 1% of penicillin/streptomycin (Welgene) in 100-pi cell culture dish (Corning) for seven days. In every other day, 5 mL of M-CSF contained media was supplemented. To harvest BM-DMs, cells were harvested by cell scraper (SPL). Purity of BM-DCs and BM-DMs were monitored with flow cytometry by CD11c, CD11b, and MHC class II expression. For the in vitro RSV infection, 2 × 10^5^ BM-DCs, or BM-DMs were stimulated with the indicated dose of live or inactivated RSV in 200 μl of 10% FBS containing RPMI 1640 media in 96 well plat bottom cell culture plate (Corning) for 18 h. 2.5 μg of CpG_2216_ (Invitrogen) was used as a stimulator in positive control experiments. Cell-free supernatants were collected, and interleukin (IL)-6 (BD Biosciences) and IFN-β (Biolegend) levels were analyzed using ELISA kits, following the manufacturer’s instructions.

### 2.5. Flow Cytometry

Single-cell suspensions of BM-DCs or BM-DMs were pretreated with anti-CD16/32 (2.4G2, BD biosciences) antibodies to block Fc receptors and stained with anti-CD11c (HL3, Biolegend), anti-CD11b (M1/70, Biolegend), CD86 (GL1, BD biosciences) and anti-MHC class II (M5/114.15.2, Biolegend) in 1% FBS in DPBS. Cells were gated based on forward and side-scatter properties to rule out cell debris and CD11c^+^ MHC class II^+^ population was regarded as BM-DCs and CD11b^+^ CD11c^-^ MHC class II^-^ population was regarded BM-DMs. Stained samples were acquired on a flow cytometer (Calibur, BD Biosciences). Final analysis and graphical output were obtained using FlowJo software (Version 9, Tree Star, Inc.).

### 2.6. Statistics

Data are presented as the mean ± standard error of the mean. Statistical significance was evaluated with two-tailed unpaired Student’s *t*-tests using Prism software (GraphPad 7.0). Differences were considered statistically significant at *p* < 0.05. 

## 3. Results

### 3.1. BM-DCs and BM-DMs Produce IFN-β in Response to RSV A2 Strain Infection

**Figure 1:**
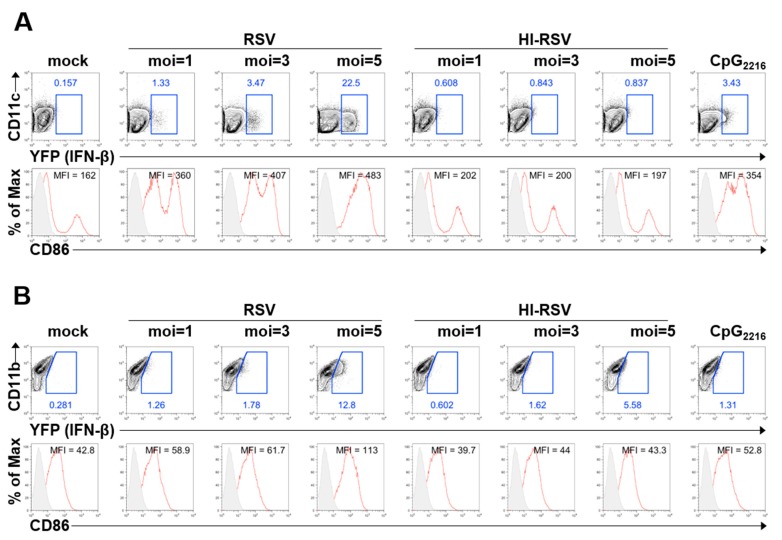
BM-DCs produce more IFN-β than BM-DMs do against RSV infection. (**A**,**B**) BM cells from IFN-β/YFP reporter mice are differentiated to BM-DCs with GM-CSF, or to BM-DMs with M-CSF. BM-DCs (A) or BM-DMs (B) were infected with the indicated dose of RSV, heat-inactivated RSV (HI-RSV), or 2.5 μg/mL CpG_2216_. These cells were harvested 18 h after stimulation and analyzed for the expression of IFN-β and CD86 by flow cytometry. The results are representative of three experiments.

Previous studies have shown that DCs and MAC cells produce type I IFN during RSV infection [21,22]. Although type I IFN is required for anti-RSV immune responses, type I IFNs are difficult to detect due to their transient expression. To overcome this problem, we analyzed the secretion of type I IFN by flow cytometry using IFN-β/YFP reporter mice. It is known that IFN-β secretion of TLR-stimulated immune cells from WT and IFN-β/YFP reporter mice is comparable [13]. To determine the IFN-β-producing capacity of DCs and MACs, GM-CSF-derived BM-DCs and M-CSF-derived BM-DMs from IFN-β/YFP reporter mice were stimulated with RSV. RSV stimulation resulted in BM-DCs and BM-DMs producing IFN-β in a viral particle dose-dependent and replication-dependent manner (Figure 1A,B), but the frequency of YFP+ BM-DMs was lower than that of BM-DCs upon RSV stimulation at the same dose.

In addition, costimulatory molecule expression was increased in RSV-stimulated BM-DCs and BM-DMs, but heat-inactivated RSV had limited ability to induce IFN-β and expression of costimulatory molecules in BM-DCs, while the induction of IFN-β is slightly increased in heat-inactivated RSV-treated BM-DMs. Taken together, we confirmed previous reports that DCs and MACs are type I IFN-producing cells on RSV infection, by using IFN-β/YFP reporter mice; additionally, we found that DCs produce more type I IFN than do MACs. Moreover, we revealed that maturation of DCs is RSV replication-dependent.

### 3.2. The MyD88- and TLR7-Mediated Pathways are Required to Induce IFN-β in BM-DCs

**Figure 2:**
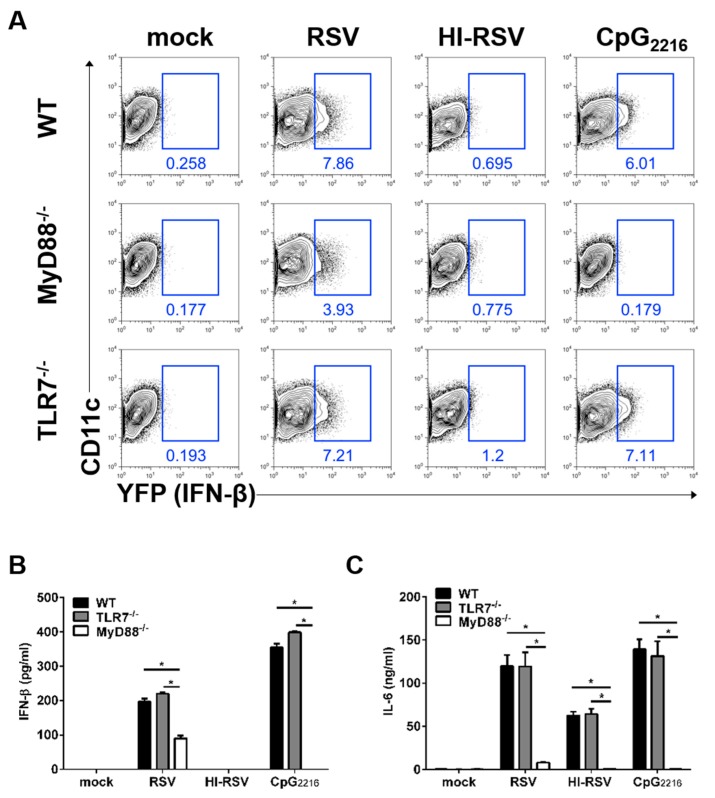
The MyD88-mediated pathways, but not TLR7-mediated pathway, are required for the production of IFN-β in BM-DCs. (**A**) BM cells from IFN-β/YFP reporter (WT) mice, MyD88-deficient IFN-β/YFP reporter (MyD88^−/−^) mice, or TLR7-deficient IFN-β/YFP reporter (TLR7^−/−^) mice were differentiated to BM-DCs. BM-DCs were infected with RSV or heat-inactivated RSV (HI-RSV) at an MOI of 3 or 2.5 μg/mL CpG_2216_. The cells were then harvested 18 h after stimulation and analyzed for IFN-β expression by flow cytometry. (**B**–**C**) Supernatants from BM-DCs were collected at 18 h after stimulation and analyzed for IFN-β (B) and IL-6 (C) by ELISA. Data are represented as mean ± SEM. The results are representative of three experiments. * *p* < 0.05 as calculated by Student’s *t*-test.

RSV can be detected by the TLR-dependent pathway in DCs [23]. In this case, TLR family proteins, such as TLR2, TLR3, TLR4, and TLR7/8 are known to sense RSV and initiate innate anti-viral immune responses in DCs. To investigate which signaling pathway drives type I IFN production in DCs in response to RSV infection, BM-DCs were generated from MyD88- or TLR7-deficient IFN-β/YFP reporter mice. MyD88-deficient, but not TLR-7-deficient, BM-DCs showed impaired induction of IFN-β production upon RSV stimulation (Figure 2A and Figure S1). 

To determine whether these data from BM-DCs reflect secreted IFN-β, we measured IFN-β and pro-inflammatory cytokine levels from these supernatants. After RSV stimulation, IFN-β secretion was impaired in supernatants from MyD88-deficient, but not TLR-7-deficient, BM-DCs (Figure 2B) and this effect was RSV replication-dependent. These results are consistent with our flow cytometric findings. The production of the pro-inflammatory cytokine IL-6 was severely affected in MyD88-deficient BM-DCs upon RSV stimulation (Figure 2C), and this effect was RSV replication-independent. Taken together, we conclude that MyD88 is required for proper innate immune responses in DCs against RSV infection, while TLR7 was found to be dispensable in DCs for RSV sensing.

### 3.3. The MyD88-Mediated Pathways are Required to Induce IFN-β in BM-DMs

**Figure 3:**
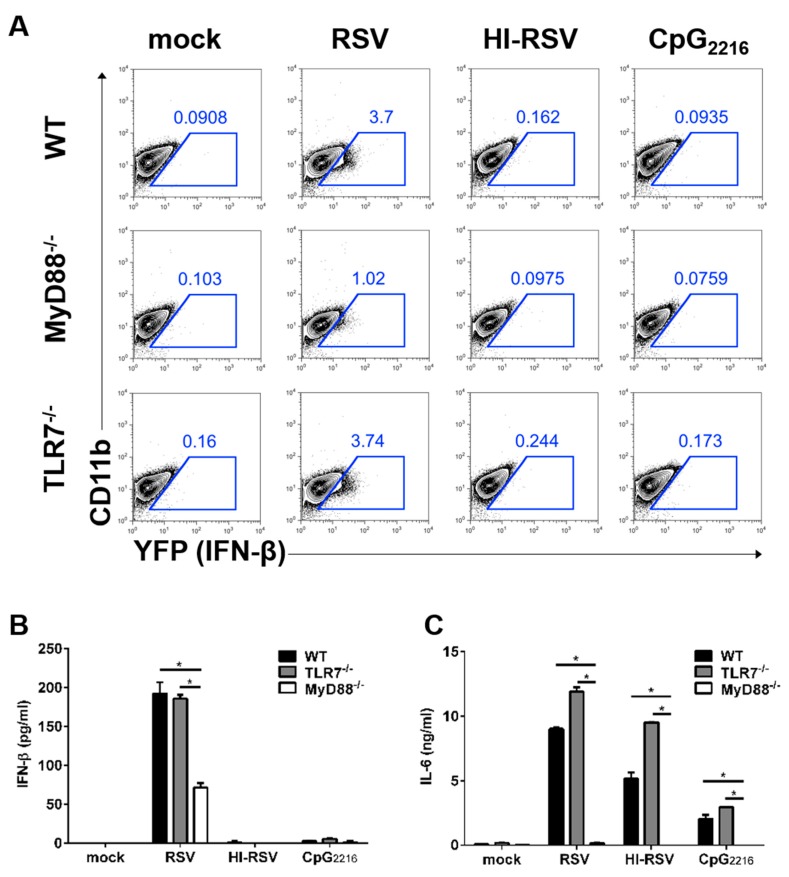
Requirement of MyD88-mediated, but not TLR7-mediated, pathways in the production of IFN-β by BM-DMs. (**A**) BM cells from IFN-β/YFP reporter (WT) mice, MyD88-deficient IFN-β/YFP reporter (MyD88^−/−^) mice, or TLR7-deficient IFN-β/YFP reporter (TLR7^−/−^) mice were differentiated into BM-DM cells. BM-DMs were infected with RSV and heat-inactivated RSV (HI-RSV) at an MOI of 3 or 2.5 μg/mL CpG_2216_. These cells were analyzed for IFN-β expression by flow cytometry 18 h after stimulation. (**B**–**C**) Supernatants from BM-DMs were collected 18 h after stimulation and analyzed for IFN-β and IL-6 by ELISA. Data are represented as mean ± SEM. The results are representative of three experiments. * *p* < 0.05 as calculated by Student’s *t*-test.

Next, we conducted experiments to investigate which signaling pathway drives type I IFN production in MACs in response to RSV infection. BM-DMs were generated from MyD88- or TLR7-deficient IFN-β/YFP reporter mice. In response to RSV infection, IFN-β production was diminished in MyD88-deficient, but not in TLR7-deficient BM-DMs (Figure 3A). 

Next, to determine whether these data from BM-DMs reflect secreted IFN-β, we measured IFN-β and pro-inflammatory cytokine IL-6 from these supernatants. After RSV stimulation, IFN-β secretion by MyD88-deficient BM-DMs decreased more than 50% (Figure 3B) but remained intact in TLR7-deficient BM-DMs. Furthermore, IFN-β production in BM-DMs was altered in an RSV replication-dependent manner. However, we found that IL-6 production was elevated in TLR-7-deficient BM-DMs following RSV infection (Figure 3C). Collectively, these results indicate that MyD88, but not TLR-7, is required for type I IFN and pro-inflammatory cytokine production in BM-DMs in response to RSV infection.

### 3.4. MAVS-Mediated Pathways are Required to Induce IFN-β in BM-DCs and BM-DMs

**Figure 4:**
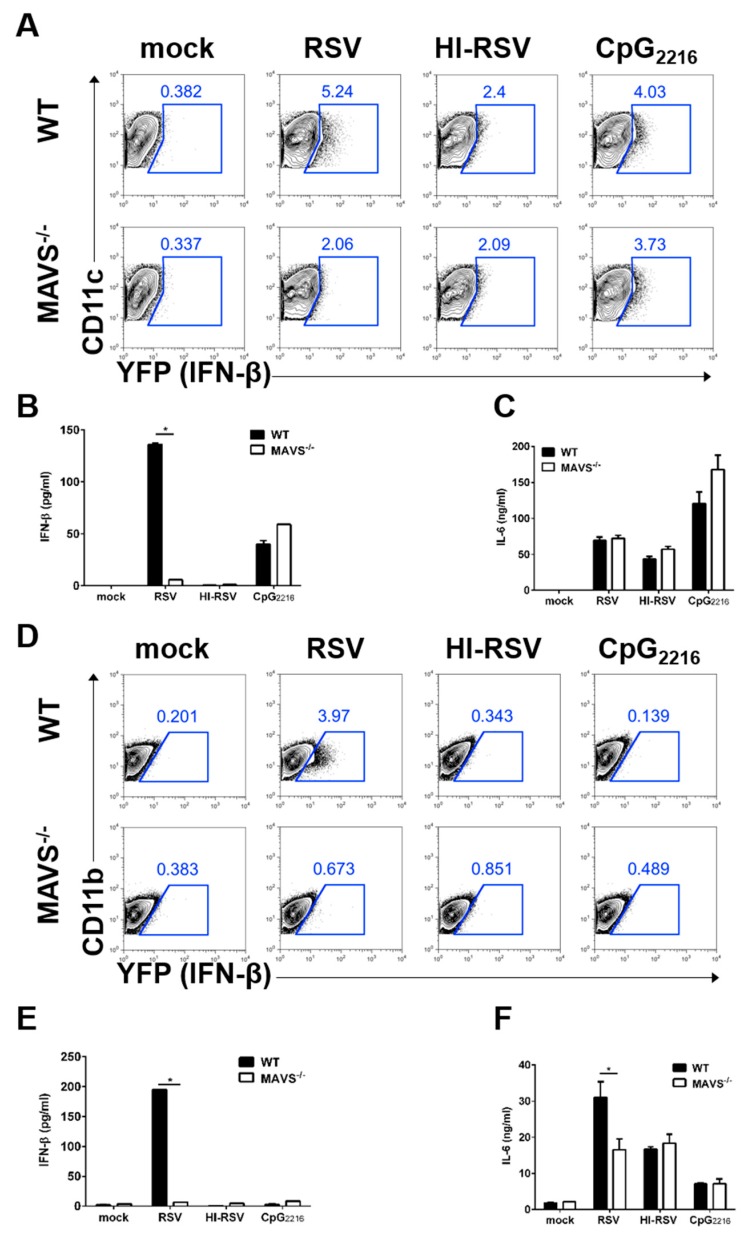
Dependency of MAVS-mediated pathways in the production of IFN-β in BM-DCs and BM-DMs. (**A**–**F**) BM cells from IFN-β/YFP reporter (WT) mice, or MAVS-deficient IFN-β/YFP reporter (MAVS^−/−^) mice were differentiated to become BM-DCs or BM-DMs. BM-DCs (A) or BM-DMs (D) were infected with RSV and heat-inactivated RSV (HI-RSV) at an MOI of 3 or 2.5 μg/mL CpG_2216_. These cells were harvested 18 h after stimulation and analyzed for IFN-β expression by flow cytometry. Supernatants from BM-DCs (B-C) or BM-DMs (E-F) were collected 18 h after stimulation and analyzed for IFN-β and IL-6 by ELISA. Data are represented as mean ± SEM. The results are representative of three experiments. * *p* < 0.05 as calculated by Student’s *t*-test.

Recent studies demonstrate that the intracellular RNA sensor RIG-I/MDA5 is required for RSV sensing and triggers innate immune responses [11,22]. Both receptors use MAVS on mitochondria for activation of downstream signal transduction. To investigate MAVS dependency in type I IFN in DCs and MACs against RSV infection, BM-DCs and BM-DMs were generated from MAVS-deficient IFN-β/YFP reporter mice. MAVS-deficient BM-DCs and BM-DMs showed impaired induction of IFN-β production upon RSV stimulation, and this effect was RSV replication-dependent (Figure 4A,D). 

Next, we measured the levels of IFN-β and pro-inflammatory cytokine IL-6 from these supernatants. Consistent with our previous flow cytometric finding, MAVS-deficient BM-DCs did not secrete IFN-β upon RSV stimulation (Figure 4B). In contrast to the IFN-β results, MAVS-deficient BM-DCs produced IL-6 at levels comparable to wild-type (WT) BM-DCs (Figure 4C). After RSV stimulation, the MAVS pathway may play a more important role in IFN-β production by BM-DMs (Figure 4E). Pro-inflammatory cytokine production was impaired in MyD88-mediated BM-DMs upon RSV stimulation, but MAVS-deficient BM-DMs could produce small amounts of pro-inflammatory cytokines, compared to WT (Figure 4F). In summary, our data showed that MAVS dependent RSV sensing is required for type I IFN production in DCs and MACs, while MAVS is necessary for the secretion of the pro-inflammatory cytokine IL-6 in MACs, but not DCs.

## 4. Discussion

In this study, we investigated the requirement for TLR7, MyD88, and MAVS in innate immune responses in DCs and MACs in response to RSV infection. Our results showed that MyD88 and MAVS play important roles in the production of type I IFN in both DCs and MACs in response to RSV infection. Type I IFN is critical for inducing lung inflammation in response to RSV infection in mice. However, type I IFNs are especially difficult to detect because of their transient expression. To overcome this problem, we utilized two reliable methods in this study. First, we used the IFN-β/YFP reporter mouse, a credible and unbiased tool for the visualization and spatiotemporal tracking of IFN-β-producing cells. We also confirmed the results of flow cytometric data at the protein level by IFN-β ELISA. These results allowed us to clearly understand whether TLR7, MyD88, and MAVS were required in the production of type I IFN and pro-inflammatory cytokine in DCs and MACs during RSV infection.

RSV predominantly infects the columnar epithelial cells of the conducting airways in the mucosal epithelium lining. Live RSV can directly infect of the various cells and activate innate immune signaling. On the other hand, adsorption of RSV virions on cell surface without infection can activate intra-cellular signaling. In DCs and MACs, several studies suggest that recognition of RSV is replication dependent [24,25].

RSV is divided into two major subtypes, RSV A and B, depending on the sequence of G glycoprotein [26]. The RSV G protein has a lot of antigenic and genetic variability, and the amino acid sequence similarity is 53% between RSV A and B [27]. Both RSV subtypes co-circulate at one or two year intervals during each RSV epidemic [28]. In this study, we focused the differential role of anti-viral sensing pathway for the production of IFN-β in DCs and MACs in A2 strain of RSV infection. Further study is needed to address the differences in immune responses in RSV strains A or B or other clinical strains. 

DCs, along with lung MACs, establish the first line of defense cell types in the innate immune response against RSV infection [29]. Recent studies show that DCs and MACs can be infected with RSV, and RSV are capable of replicating in these cells [30,31]. DCs and MACs express TLRs and RIG-I/MAVS, and several studies demonstrate the roles of these PRRs in RSV infection [7]. However, the identity of the specific TLR or RIG-I/MAVS pathway that is predominantly important for RSV infection in DCs or MACs is not yet known. We found that MyD88, a central player in innate immune signaling, is required for type I IFN and pro-inflammatory cytokine production in response to RSV infection in DCs and MACs. Although MyD88 is critically required for innate and adaptive immune responses against RSV infection [22,32,33], it is not known that which viral factors of RSV are associated with Myd88 protein related signaling pathway and further study is needed for finding their relationship. 

TLR7 may not be required during RSV infection. This result is consistent with a previous finding that TLR7 deficiency has no effect on type I IFN production in RSV infection [10]. Furthermore, our data confirmed the results of a previous study that MyD88 is essential for the innate immune response in RSV infection [22]. Although RSV, a single-strand negative sense RNA virus, is mainly recognized by endosomal TLR7, other TLRs have been reported to be involved in RSV sensing [34]. Our study suggests that other TLRs may be associated with type I IFN secretion during RSV infection because MyD88 is involved in other TLRs signal cascades for innate immune responses. 

Activated RLRs then signal through MAVS to induce the production of type I IFNs [35]. MAVS is expressed on every nucleated cell and can produce type I IFNs via the cytosolic detection pathway. Recently, papers show that lung DCs or alveolar MACs are the major source of type I IFN in RSV infection and AMs detect RSV via RIG-I/MAVS [11,36,37]. We found that MAVS protein is important for type I IFN production in DCs and MACs in response to RSV infection. However, we found that MAVS deficiency does not significantly impair pro-inflammatory cytokine IL-6 production in DCs. MAVS induces the phosphorylation of IRF3 and IRF7 and NF-κB activation leading to the production of type I IFNs and pro-inflammatory cytokines, respectively. A study shows that RSV Nonstructural Protein 1 (NS1) associates with MAVS in RSV infection and disrupts MAVS interaction with RIG-I, as well as type I IFN production [38]. This means that the RSV NS1 protein inhibits MAVS-dependent type I IFN responses. Furthermore, a recent report indicated that NF-κB activation was substantially lower in cells infected with Nonstructural Protein 2 (NS2)-deficient or NS1/2-deficient RSV [39]. This result suggests that NS2 is involved in NF-κB activation in RSV infection. In summary, RSV may be differentially regulated by type I IFN and pro-inflammatory cytokines. However, to gain a more comprehensive understanding, future studies will need to investigate the pathway for the secretion of type I IFN or pro-inflammatory cytokines in MAVS-dependent RSV sensing in DCs. Additionally, further studies will be needed to understand the relative contribution of different PRRs to the activation of DCs or MACs in RSV infection. 

Our type I IFN detection system was able to effectively observe the secretion of type I IFN in DCs and MACs in RSV infection. However, our study did not investigate type I IFN secretion in DCs and MACs in lungs in vivo. A similar approach, using Ifna6gfp/+ mice, has been conducted to identify the source of type I IFN in RSV infection [11]. However, α and β IFN exhibit key differences in several biological properties [40]. Future studies are needed to confirm differences between IFN-α and IFN-β secretory cells in RSV infection in vivo.

In conclusion, using IFN-β visualization, we found that MyD88 and MAVS, but not TLR7, are important for RSV sensing and type I IFN production in DCs and MACs. Our results can be applied to the development of novel RSV vaccines.

## Supplementary Materials

The following are available online at http://www.mdpi.com/1999-4915/11/1/62/s1, Figure S1: The MyD88-dependent pathways, but not TLR7-dependent pathway, are essential for the production of IFN-β in BM-DCs.
