# Supplementary material for: Differential Role of Anti-Viral Sensing Pathway for the Production of Type I Interferon β in Dendritic Cells and Macrophages Against Respiratory Syncytial Virus A2 Strain Infection
Source: Viruses. 2019 Jan 15;11(1):62. doi: 10.3390/v11010062 (PMC6356365; doi:10.3390/v11010062)
Supplement: Supplementary file 1 [file viruses-11-00062-s001.pdf]

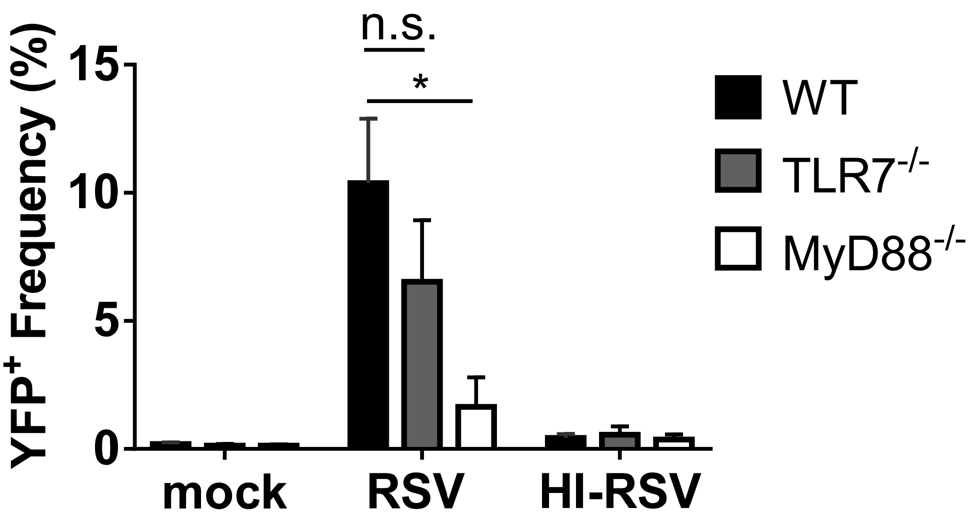


**Figure S1.** The MyD88-dependent pathways, but not TLR7-dependent pathway, are essential for the production of IFN-β in BM-DCs. (A) BM cells from IFN-β/YFP reporter (WT) mice, MyD88-deficient IFN-β/YFP reporter ($MyD88^{-/-}$) mice or TLR7-deficient IFN-β/YFP reporter ($TLR7^{-/-}$) mice were differentiated to BM-DCs. BM-DCs were infected with RSV and heat-inactivated RSV (HI-RSV) at an MOI. These cells were harvested at 18 h after stimulation and analyzed for the expression of IFN-β by flow cytometry. Frequency of $YFP^{+}$ cells shown as bar graph. Data were combined from three biological experiments and data are represented as mean ± SEM.

Oh et al, supplement figure 1
